# 2029 Surface display of chimeric proteins for exosome imaging and capturing in mammalians

**DOI:** 10.1017/cts.2018.124

**Published:** 2018-11-21

**Authors:** Mai A. Do, Daniel H. Levy, Stacie Lim, Natalie Duong, Kevin Curley, Grace Ling, Biao Lu

**Affiliations:** Santa Clara University, Santa Clara, CA, USA

## Abstract

OBJECTIVES/SPECIFIC AIMS: Exosomes are living nanoscale vesicles that can shuttle large amounts of bioactive cargo for intercellular communication. The potential of these nanovesicles to serve as both biomarkers for disease diagnosis and vehicles for delivery of therapeutics has only begun to be explored. To realize these potentials, molecular tools for effective exosome tracking and capturing must be invented in order to advance basic research and clinical translation. METHODS/STUDY POPULATION: We utilize a surface display strategy that enables exosome modification in living mammalian systems. By reconfiguring the surface protein CD63 or viral envelope glycoprotein VSV-G, we generate 3 topologically distinctive protein chimeras for exosome imaging and capture in mammalian systems. RESULTS/ANTICIPATED RESULTS: We have shown that these genetically encoded protein chimeras have the ability to correctly target and integrate into exosomes in cultured human cells. Furthermore, we have demonstrated that the secreted exosomes could be successfully captured by an affinity peptide intentionally displayed on the outer surface of exosomes. DISCUSSION/SIGNIFICANCE OF IMPACT: Our study highlights the potential of these fusion proteins for exosome tracking and provides novel genetic tools for exosome research and translation, one of which is loading protein therapeutics for targeted delivery.

